# *Haemaphysalis hoodi* (Acari: Ixodidae) on a human from Yaoundé, Cameroon, and its molecular characterization

**DOI:** 10.1007/s00436-022-07613-5

**Published:** 2022-08-05

**Authors:** Archile Paguem, Ben J. Mans, Manchang Kingsley, Alfons Renz, Dmitry A. Apanaskevich, Lidia Chitimia-Dobler

**Affiliations:** 1 Department of Comparative Zoology, Institute of Evolution and Ecology, University of Tübingen, 72076 Tübingen, Germany; 2 Department of Veterinary Medicine, Faculty of Agriculture and Veterinary Medicine, University of Buea, Buea, Cameroon; 3 Epidemiology, Parasites and Vectors, Agricultural Research Council-Onderstepoort Veterinary Research, Onderstepoort, South Africa; 4 The Department of Veterinary Tropical Diseases, University of Pretoria, Pretoria, South Africa; 5 Department of Life and Consumer Sciences, University of South Africa, Pretoria, South Africa; 6 United States National Tick Collection, The James H. Oliver, Jr. Institute for Coastal Plain Science, Georgia Southern University, Statesboro, GA 30460-8042 USA; 7 Department of Biology, Georgia Southern University, Statesboro, GA 30460 USA; 8 Zoological Institute, Russian Academy of Sciences, St. Petersburg, 199034 Russia; 9 Bundeswehr Institute of Microbiology, Neuherbergstrasse 11, 80937 Munich, Germany

**Keywords:** Human, Tick, *Haemaphysalis hoodi*, Cameroon

## Abstract

The genus *Haemaphysalis* Koch, 1844 (Acari: Ixodidae) is the second-largest genus, with more than 170 described species that primarily parasitize mammals and birds (Guglielmone et al. 2014, Guglielmone et al. 2020). *Haemaphysalis* species are three-host ticks, mainly distributed in southern and southeastern Asia and tropical Africa (Guglielmone et al. 2014). The present study identified a tick, *Haemaphysalis hoodi* Warburton & Nuttall, 1909, collected from a human in Yaoundé, Cameroon. This tick species feed on birds in sub-Saharan Africa. To the best of our knowledge, this is the second record of *H. hoodi* from humans. In addition, 16S ribosomal RNA and cytochrome oxidase I sequences were generated for this species for the first time. Screening pan-*Rickettsia*-PCR infection gave a negative result.

## Introduction

The genus *Haemaphysalis* Koch, 1844 (Acari: Ixodidae) is the second-largest genus, with more than 170 described species (Guglielmone et al. 2020). *Haemaphysalis* species are three-host ticks that primarily parasitize mammals and birds (Guglielmone et al. 2014). Species from this genus are mainly distributed in southern and southeastern Asia and tropical Africa, some species are known from Australia, and only a few species occur in the Americas (Guglielmone et al. 2014). *Haemaphysalis* species are reservoirs and vectors of many pathogenic microorganisms of animals and humans. For instance, *Haemaphysalis leachi* (Audouin, 1826) transmits *Babesia rossi* in dogs (Kamani 2021), as well as *Rickettsia conorii*, which causes human tick-bite fever, and *Coxiella burnetii*, the causative agent of Q fever (Hoogstraal 1956). Limited studies have been performed on *Haemaphysalis* species from wildlife that focused on their diversity and role as potential vectors and reservoirs of pathogens.

Out of 47 described *Haemaphysalis* species endemic to the Afrotropic region, eleven species are known from Cameroon, namely *H. aciculifer* Warburton, 1913, *H. camicasi* Tomlinson & Apanaskevich 2019, *H. hoodi* Warburton & Nuttall, 1909, *H. houyi* Nuttall & Warburton, 1915, *H. leachi*, *H. moreli* Camicas et al. 1972, *H. paraleachi* Camicas et al. 1983, *H. parmata* Neumann, 1905, *H. princeps* Tomlinson & Apanaskevich 2019, *H. punctaleachi* Camicas et al. 1973, and *H. tauffliebi* Morel 1965 (Morel and Mouchet 1958; Morel 1965; Camicas et al. 1972, 1973, 1983; Hoogstraal and El Kammah 1972; Apanaskevich et al. 2007; Tomlinson and Apanaskevich 2019). The haemaphysalid subgenus *Ornithophysalis* Hoogstraal & Wassef, 1973, comprises 19 species divided into five structural-biological groups (Hoogstraal and Wassef 1973; Camicas et al. 1998). Many of the species have not been adequately studied structurally, biologically, or epidemiologically (Hoogstraal and Wassef 1973). *Haemaphysalis hoodi* is one of the four species of the *Haemaphysalis doenitzi* group, which also includes *H. doenitzi* Warburton & Nuttall, 1909, *H. phasiana* Saito, Hoogstraal & Wassef, 1974, and *H. madagascariensis* Colas-Belcour & Millot, 1948 (Camicas et al. 1998). This species is broadly distributed in sub-Saharan Africa (Hoogstraal 1956; Hoogstraal and Wassef 1973). Adults, nymphs, and larvae of *H. hoodi* feed primarily on various groups of birds, while records from mammals are rare (Guglielmone et al. 2014). In Cameroon, *H. hoodi* was recorded from different ground-feeding bird species (Hoogstraal 1956; Santos Dias 1958). Here, we report for the first time a specimen of this species collected from a human in Yaoundé, Cameroon, and provide data on its mitochondrial (16S rRNA, cox I) genes.

## Material and methods

In late October 2021, a light brown tick was removed manually from the shoulder of a woman in Nkozoa in the Mefou and Afamba Division of the center region of Cameroon (3°52′53.2″N 11°41′54.6″E). The collected tick was transferred to a 1.5 ml tube containing 600 µl absolute ethanol and sent to Bundeswehr Institute of Microbiology, Munich, Germany, for investigation. The tick specimen was first identified using morphological keys (Hoogstraal 1956), under a Keyence VHX‑900F microscope (Itasca, IL, USA). DNA was extracted using the QIAamp mini DNA extraction kit (Qiagen, Hilden, Germany) according to the manufacturer’s instructions. As this is a rare tick species and no sequences from this species are available, 16S rRNA (Halos et al. 2004) and cox I (Apanaskevich et al. 2011) mitochondrial genes were sequenced, and the sequences obtained were edited and compared with the respective sequences deposited in GenBank using BLASTN and phylogenetic analysis. Sequences for each gene were aligned using MAFFT (Katoh and Standley 2013) with default parameters and phylogenetic analyses performed with IQ-Tree2 v1.6.12 (Minh et al. 2020). Optimal evolutionary models were calculated for each gene: 16S (K3Pu + F + I + G4) and Cox1 (TIM2 + F + I + G4). Nodal support was estimated using ultrafast bootstrap (*n* = 10,000) and the 50% consensus trees were reported. The partial sequences of the mitochondrial 16S rRNA and cox I genes generated in this study for *H. hoodi* species have been deposited in GenBank under the accession numbers ON189038 and ON191014. Additionally, *Rickettsia* spp. screening was performed using a previously published real-time PCR assay targeting a part of the *gltA* gene (Wölfel et al. 2008).

## Results and discussion

**Fig. 1:**
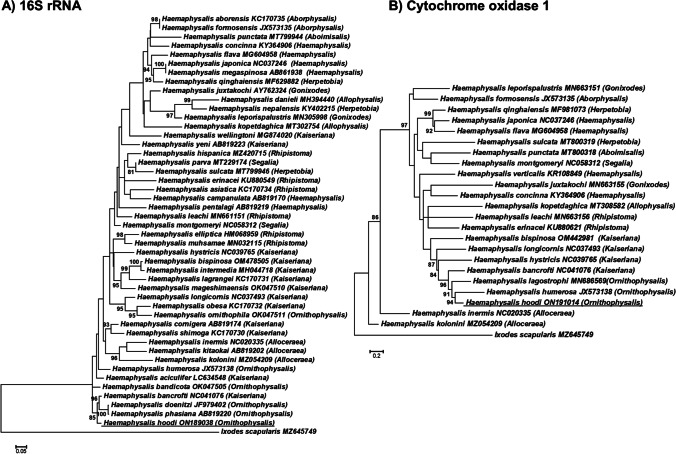
*Haemaphysalis hoodi* female collected from a human in Cameroon: **A** dorsal view, **B** ventral view

The tick was identified as a female *Haemaphysalis hoodi*. The specific characteristics of the female include moderately dense punctations on scutum, broadly salient palpi, and absence of posterodorsal spur on palpal segment II (Fig. 1A,B) (Hoogstraal 1956; Morel 1965). Birds, especially ground feeder birds, are specific hosts for species within the *Ornithophysalis* subgenus; although some species parasitize birds and mammals, others only parasitize mammals (Hoogstraal 1956; Hoogstraal and Wassef 1973).

**Fig. 2:**
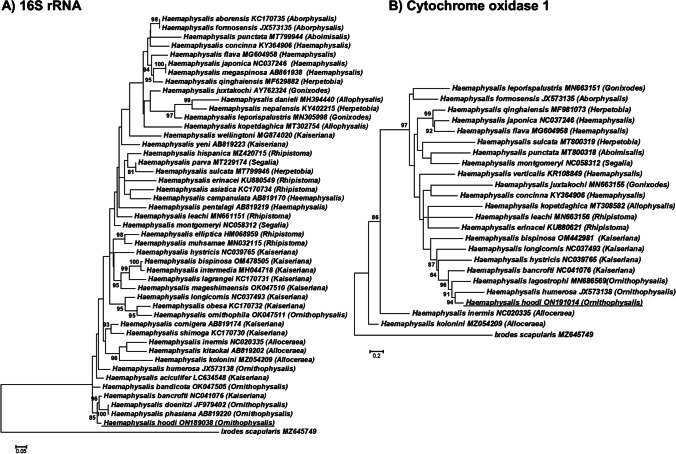
Maximum likelihood analysis of the 16S rRNA and cox I genes for the genus *Haemaphysalis*. Bootstrap support above 80% is indicated and the trees were rooted with *Ixodes scapularis*. The accession numbers used for the 16S rRNA and cox I genes are indicated behind the species names, respectively, and the tick sequenced in the current study is underlined. Subgenera are indicated in parentheses

*Haemaphysalis hoodi* is a very rare parasite of humans (Guglielmone and Robbins 2018). Adults of *H. hoodi* have been found in three cases of human infestation in Ivory Coast although the exact localities were not reported (cited in Guglielmone et al. 2018). Our finding represents the second record of this tick species feeding on humans. Phylogenetic analysis indicated that the 16S rRNA sequence obtained (305 bp) from *H. hoodi* group in a moderately supported clade with *H. bancrofti*, *H. doenitzi*, and *H. phasiana* (Fig. 2). Of interest is that *H. bancrofti* is also part of this clade since it is classified in the subgenus *Kaiseriana* Dias, 1963, while *H. hoodi* and *H. phasiana* are classified in the *Ornithophysalis* Hoogstraal and Wassef, 1973, subgenus (Hoogstraal and Wassef 1973). Similarly, in the cox I analysis (636 bp), *H. hoodi* group in a well-supported clade with *H. bancrofti*, *H. humerosa*, and *H. lagostrophi*, the latter two species also belonging to the subgenus *Ornithophysalis* (Hoogstraal and Wassef 1973). While the overall support for the trees was weak, in each tree, several clades with good bootstrap support were obtained. No overwhelming support was found for any of the subgenera as monophyletic lineages. This may be due to limited phylogenetic signal due to the short sequences used in the analysis. It may, however, be noted that a recent analysis using 10 mitochondrial genes also resulted in a paraphyletic *Haemaphysalis* subgenus (Kelava et al. 2021). As such, more studies that focus on molecular systematics of the *Haemaphysalis* subgenera are needed to ascertain the validity of various subgenera. Even so, both 16S rRNA and cox I indicate that *H. hoodi* presents a unique genetic signature compared to other sequences available in the database that shows a genetic relationship to other members of the *Ornithophysalis* subgenus.

*Rickettsia* spp. DNA was not amplified in the sample obtained from the *H. hoodi* female. It would have been expected to detect *Rickettsia africae*, which is responsible for the African tick-bite fever, mainly transmitted by *Amblyomma* species or *Rickettsia aeschlimannii*, a *Hyalomma* species related ricketsiae. *Haemaphysalis* species from Africa are not known as vectors for *Rickettsia* species. *Haemaphysalis leachi* was supposed to be a vector for *Rickettsia conorii* in southern Africa, but no isolates are available to confirm this. In Asia, especially in China, many *Haemaphysalis* species are vectors for *Rickettsia* spp., e.g., *Rickettsia sibirica*, *Rickettsia heilongjiangensis*, and *Rickettsia japonica* (Raoult and Parola 2007).

## Data Availability

The sequences were submitted to GenBank under the following access numbers: ON189038, ON191014. The tick specimen is in the LCD collection at Bundeswehr Institute of Microbiology.
