# Correction to: Haemaphysalis hoodi (Acari: Ixodidae) on a human from Yaoundé, Cameroon, and its molecular characterization

**DOI:** 10.1007/s00436-022-07625-1

**Published:** 2022-08-26

**Authors:** Archile Paguem, Ben J. Mans, Manchang Kingsley, Alfons Renz, Dmitry A. Apanaskevich, Lidia Chitimia‑Dobler

**Affiliations:** 1 Department of Comparative Zoology, Institute of Evolution and Ecology, University of Tübingen, 72076 Tübingen, Germany; 2 Department of Veterinary Medicine, Faculty of Agriculture and Veterinary Medicine, University of Buea, Buea, Cameroon; 3 Epidemiology, Parasites and Vectors, Agricultural Research Council-Onderstepoort Veterinary Research, Onderstepoort, South Africa; 4 The Department of Veterinary Tropical Diseases, University of Pretoria, Pretoria, South Africa; 5 Department of Life and Consumer Sciences, University of South Africa, Pretoria, South Africa; 6 United States National Tick Collection, The James H. Oliver, Jr. Institute for Coastal Plain Science, Georgia Southern University, Statesboro, GA 30460‑8042 USA; 7 Department of Biology, Georgia Southern University, Statesboro, GA 30460 USA; 8 Zoological Institute, Russian Academy of Sciences, St. Petersburg, 199034 Russia; 9 Bundeswehr Institute of Microbiology, Neuherbergstrasse 11, 80937 Munich, Germany

**Correction to** **:** **Parasitology Research**

**https://doi.org/10.1007/s00436-022-07613-5**

The authors regret that Figure 1 and Figure 2 were swapped incorrectly in the published version of the article. The original article has been corrected.

